# Exploring Psychosocial Needs of Patients with Cancers through the Lens of the Physicians and Nurses: A Qualitative Study

**DOI:** 10.1155/2024/2175517

**Published:** 2024-04-22

**Authors:** A. Fernández-Feito, C. Alonso-Iglesias, M. Paz-Zulueta, A. Pellico-López

**Affiliations:** ^1^Área de Enfermería, Facultad de Medicina y Ciencias de la Salud, Universidad de Oviedo, Avda. Julián Clavería s/n, Oviedo 33006, Spain; ^2^Instituto de Investigación Sanitaria del Principado de Asturias (ISPA), Grupo de Investigación en Cuidados, Av. del Hospital Universitario, 33011 Oviedo, Spain; ^3^Facultad de Medicina y Ciencias de la Salud, Universidad de Oviedo, Avda. Julián Clavería s/n, 33006 Oviedo, Spain; ^4^Departamento de Enfermería, Universidad de Cantabria, Avda Valdecilla s/n. C.P, 39008 Santander, Spain; ^5^Instituto de Investigación Sanitaria Valdecilla (IDIVAL), Grupo de Investigación en Derecho Sanitario y Bioética (GRIDES), C/Cardenal Herrera Oria s/n, 39011 Cantabria, Spain; ^6^Servicio de Salud de Cantabria, Avda. Derechos de la Infancia 31, 39340 Cantabria, Spain

## Abstract

**Aims:**

To explore the experience of nurses and physicians regarding psychosocial needs of patients with cancer and to describe their perception according to professional category and clinical setting.

**Design:**

A qualitative descriptive study.

**Method:**

14 nurses and 12 physicians were selected from three hospital clinical units and four primary care centers in northern Spain. Data were collected using semistructured interviews. Content analysis was performed using open coding. Reporting of findings followed the COREQ checklist.

**Results:**

Four themes were identified: the needs of patients with cancer, psychosocial care provided by health professionals, difficulties addressing psychosocial needs, and available resources. According to nurses and physicians, being diagnosed with cancer involves a radical life-changing process, with a profound impact at the psychosocial level. Within the field of psychosocial care, the role perceived by each profession was different. Thus, nurses highlighted the need for these patients to receive emotional support and care, although limited importance is given to psychosocial needs. The role of physicians was more focused on referring these patients to other health professionals. The lack of training or time was one of the main difficulties perceived by the professionals. The family is a fundamental resource and, overall, patients are provided with limited information about other psychosocial resources. At the hospital, the emphasis is placed on physical needs and health professionals experience greater burnout and fear of compassion fatigue. Primary care teams could, therefore, have a primary role in addressing psychosocial needs due to their understanding of each person's context and personal circumstances.

**Conclusions:**

It is essential for nurses and physicians to consider the psychosocial needs of patients with cancer. However, these needs are not always adequately addressed. Further resources are required to reduce the workload, increase the training of health professionals, and introduce organizational changes to consider psychosocial needs during routine care.

## 1. Introduction

The importance of integrating psychosocial care within the routine care of patients with cancer has been increasingly recognized [1], especially as numerous unmet psychosocial needs (PNs) have been identified [2], which can have a negative impact on their quality of life [2, 3].

Previous studies have shown that unmet PNs affect oncology patients with different pathologies such as hematological tumors [4], gynecological cancer [5], or colorectal cancer [3, 6]. These PN are related to information needs [2, 3, 7], fear of recurrence [3], support needs [5, 7], or psychological issues in general [2]. However, it is also known that patients do not always express their unmet PNs, sometimes for fear of stigmatization or because of difficulties encountered in the physician-patient-relationship [8]. And as expressed by oncology nurses, this can be a problem especially in older cancer patients where the needs may go unnoticed [9].

In psychosocial care, health professionals play a fundamental role. Specifically, nurses play an important role in identifying and meeting unmet psychosocial needs in different contexts, such as clinical units before and after surgery, oncology units, and/or primary care, considered an essential element in the provision of psychosocial care [9]. However, for oncology nurses working in an emotionally charged environment and with time constraints, despite recognizing the importance of PNs, their priority was physical needs, as providing psychosocial care is often a secondary concern, based on the limited time available [10].

Furthermore, this role may be perceived as a challenge, in relation to the particularities of cancer and patient characteristics, because of the lack of support from the team or the institution. Thus, a former study found that providing psychosocial care was an overall satisfying process for nurses, allowing them to grow as individuals, despite coming at a high cost in terms of fatigue and emotional exhaustion [11]. Moreover, there is limited evidence on the role of nurses in addressing PNs in cancer patients in surgical units or in primary care teams [12].

Regarding physicians' response to PNs, according to the perspective of primary care physicians, they identified that their role should be to coordinate patient care, manage comorbidities, and provide information and psychosocial support to patients and their families. However, the role of these primary care professionals is not always close to this ideal, mainly due to communication problems that derive in a lack of updated reports or a lack of clarity regarding their role [13]. These findings are generally consistent with another study on the barriers encountered by primary care physicians caring for cancer patients, which included limited knowledge, lack of time or financial incentives, or less patient trust [14].

Some of the proposals to improve the ability to address these PNs, with considerable consensus among patients and healthcare professionals, would be to change certain behaviors to improve the support provided by the clinical staff and the availability of resources [8]. It would also be advisable to routinely ask patients about their needs and expectations, especially in some patients who may have difficulties in identifying and expressing their demands, e.g., due to advanced age [9].

In terms of training needs, it has been proposed that all primary care providers should be trained to care for the growing number of cancer patients, maintaining good communication and working together with oncologists. These training needs refer to both initial training and continued education throughout their careers, in order to research and implement evidence-based guidelines and assess quality of life in cancer survivors [15].

Therefore, it is still necessary to investigate the involvement of the different health professionals in the approach to PNs, considering the importance of implementing psychosocial assessments in routine care [16]. It is important to incorporate their point of view, since most studies have been carried out from the perspective of patients and/or family members [2, 7] and the perception of health professionals such as cancer surgeons or medical residents is unknown, whose experience has been less explored. In addition, it is becoming increasingly important to address psychosocial demands through multidisciplinary teams [17] considering the main settings (hospital and primary care) where these patients with cancer receive care for years.

The aims of this study were to explore the experience of nurses and physicians regarding psychosocial needs in patients with cancer and to describe their perception according to professional category (nurses/physicians) and clinical setting (hospital/primary care).

## 2. Methods

### 2.1. Design

A descriptive qualitative study was conducted. The rationale for this type of research is based on the interest in collecting the subjective and close-up views of health professionals caring for cancer patients in the real clinical setting. The aim is to obtain a straight description of the phenomena, trying to understand their experience in their context in order to carry out an analysis and interpretation of the findings while staying close to the data [18, 19].

### 2.2. Participants and Settings

This study included nurses and physicians from three clinical units (oncology, general surgery, and otorhinolaryngology surgery) of the Hospital Universitario Central de Asturias (level 3 hospital with about 1000 beds) and from four primary care centers in two urban health districts in Asturias (Northern Spain). The inclusion criteria were being a nurse, physician, or resident physician, being active (i.e., not being on medical leave), and with a minimum of two years of clinical professional experience caring for cancer patients in the hospital or in primary care (not exclusively oncology units). The exclusion criteria were a personal history of cancer, with academic qualifications other than nursing or medicine (psychology and social work), and working in the oncology day hospital (chemotherapy administration center) or the radiotherapy services. Professionals with a heterogeneous profile in terms of sex, age, and professional experience were included in the study applying the following segmentation criteria: profession (nurse/physician) and clinical setting (hospital/primary care).

Purposive sampling was used to select healthcare professionals, either through the research team's contacts or through the involvement of gatekeepers such as the supervisor of the hospital oncology unit. In addition, snowball sampling was used, where participants proposed new health professionals that were relevant to the study. Finally, 26 health professionals (14 nurses and 12 physicians) participated in the study. None of the people contacted refused to participate in the study. Each health professional was contacted by telephone at their workplace, to explain the study purpose and methodology, together with the voluntary and confidential nature of their participation. The respondents did not receive any compensation for their participation and were informed of the independent nature of this research in relation to their workplace.

### 2.3. Individual Interviews

Data collection took place via individual semistructured interviews. An interview script was designed, which was previously agreed upon by the work team, mainly addressing the needs of cancer patients, the psychosocial assessment of these patients, and proposals for improving psychosocial care (Table 1).

First, a pilot interview was conducted with the nursing supervisor of the oncology unit of the Hospital Universitario Central de Asturias. The research team who conducted the interviews consisted of a female nursing student who based her final degree project on this study, a social worker (female) with experience in health social work, and a university professor (female) with experience in nursing care research and qualitative methodology. Only the university professor was previously acquainted with some of the participants for work and teaching-related reasons. Both an interviewer and an observer were present during the interviews.

The interviews took place between January and March 2020 in the professionals' workplaces before starting or at the end of their working day, according to the participants' wishes, all of which were audio-recorded. All participants were informed of the study objective and the characteristics of the interviewers (profession and institutions involved in the study). A data collection sheet was used for each professional (sex, age, professional profile, unit, and work center) together with a field diary. The diary was used to compile the characteristics of the meeting (place, duration, consent, voice recording) and a summary of the interview content, as well as other relevant aspects of nonverbal communication, specific cases during their professional experience, etc. The duration of each interview ranged between 10 and 30 minutes. The criterion for terminating the interviews was saturation of the discourse. No interviews had to be repeated.

Each participant was offered the possibility of listening to the recording or reading the interview transcript. Only one nurse requested to review her interview, which was sent to her by e-mail; however, she made no changes to the content.

### 2.4. Data Analysis

The analysis was carried out in three main phases: preparation of the data, organization of results, and reporting, according to Elo and Kyngäs [20]. In the preparation phase, each complete interview was established as the unit of analysis, establishing that only the manifest content from the verbatim transcription of the interviews would be analyzed, without considering nonverbal language (silences, posture, etc.). Next, an inductive content analysis was conducted, where themes emerged from the data through open coding. Two independent analysts selected two interviews from two participants with a different profile (nurse in a surgical unit and a palliative physician in primary care) to establish the coding paradigm. To familiarize themselves with the material, each interview was read several times. The intersubjectivity of the team was carried out through the interactive work of the researchers, enriching the analytical capacity and providing a deeper understanding, as opposed to studies where a lone researcher performs the analysis. The contributions of each researcher were valued by the rest of the researchers during the team discussions, with an open mind that subsequently stimulated reflection. The coding system was agreed upon by both researchers, calling on the third researcher in the case of discrepancies. Subsequently, a different analyst completed the coding of all interviews. The codes were grouped into categories that emerged freely in this phase. These categories were then grouped into a higher hierarchical system, organized into themes. When several categories referred to a similar content, they were collapsed. Each category was named using content-characteristic words. Themes were refined in successive rounds of the analysis after gaining a better understanding of the meaning of the nurses' and physicians' experience of PNs. Regarding the last phase, the results were shown in a table, presenting the classification system of the data in codes, subthemes, and topics. The most representative, authentic citations were included in the Results section along with the main themes and subthemes. This helped to increase the trustworthiness of the research, by pointing out to readers what kind of original data lead to the formulated categories. The results were sent to three nurses as a feedback measure with the participants. Throughout the process, the researchers maintained a reflective attitude, being aware of how their thoughts and experiences fed into the analysis, which, in turn, led to changes in themselves as a result of the research process [21]. Nonetheless, they were able to maintain the necessary distance as researchers to see beyond the participants' narratives, as well as contradictions and problems related to their contributions [21].

The rigor of the research is fundamentally based on the clear sample rationale and the triangulation of researchers during the data analysis [22].

For the processing and organization of the data, we used MAXQDA software. The study findings were reported according to the consolidated criteria for reporting qualitative research (COREQ) [23].

### 2.5. Ethical Considerations

Favorable permission was obtained from the Clinical Research Ethics Committee of Principado of Asturias (REF 314/19) and the Directorate of Nursing of the health district. All participants signed the informed consent form. The study complied with local regulations for the approval of observational studies in Europe and according to the Helsinki Declaration.

## 3. Results

### 3.1. Participant Characteristics

The characteristics of the 26 participants are described in Table 2. The distribution by sex was balanced, with 53.8% women. The mean age was 48.1 years, ranging from 26 to 61 years. In total, 14 nurses and 12 physicians were included. Over half of the professionals (61.5%) worked at the hospital, distributed among three services: two surgical (surgery and otorhinolaryngology) and one medical service (oncology). The remaining professionals worked in primary care, which also included a physician and a nurse from the palliative home care unit. The mean experience was 22.3 years, ranging from 2 to 43 years. Only six professionals (four nurses and two physicians) had training in addressing psychosocial needs. Sixteen professionals had family members or friends with cancer in their close environment.

### 3.2. The Experience of Nurses and Physicians regarding Psychosocial Needs in Patients with Cancer

No pre-established coding system was established; therefore, the ideas or concepts emerged directly from the data through an inductive analysis. The information was segmented and grouped into four levels of coding (subcodes, codes, subthemes, and themes). Four themes were identified: the needs of patients with cancer, the psychosocial care provided by health professionals, the difficulties in addressing psychosocial needs, and the resources available.

Table 3 describes the needs of patients with cancer according to nurses and physicians. Within this theme, 24 codes and six subthemes were identified. The professionals recounted that these patients need psychological or emotional support throughout the process, although some reject it at first because they feel strong and believe they do not need it. Virtually, all patients have important emotional needs (love, affection, empathy, and feeling supported) because facing cancer reveals their emotional weaknesses. In the process of coming to terms with the diagnosis, taking antidepressants is not always helpful. It is also important to pay attention to how emotions change at different stages after receiving the diagnosis; i.e., emotions change over time, and each patient has a unique emotional response that they may or may not show.

Although they associate having cancer with a loss of functionality and independence, the needs of these patients transcend physical symptoms, and the psychosocial sphere becomes even more of a priority than the physical sphere. They need help to cope with the most frequent symptoms such as pain or fatigue but also to physically adapt to living with the disease and its chronic sequelae.

They also need to receive information, but it is important to consider each person's wishes about how much they want to receive.

People with cancer want to talk, share, and verbalize their fears, especially regarding facing death or overcoming the social stigma attached to having cancer. These patients demand to be listened to, as they are sometimes unable to vent their feelings to their families and expect healthcare professionals to allow them to express themselves without reservation. They often want to talk about their feelings, about hopelessness in the final stages, about unfulfilled dreams, and about decisions they would like to make, and sharing these thoughts and emotions helps them to cope.

Many professionals likened a cancer diagnosis to a tsunami that forces patients to face uncertainty with difficulty to accept the reality and/or the diagnosis.

“Then they have to adapt their life, reorganize it. There are other people who can't come to terms with this situation, the change that comes first with the diagnosis and then the symptoms, because they need help with basic day-to-day activities, depending on what the disease may cause, and then to adapt their life” (oncologist, hospital).

Finally, these patients require a lot of care that usually starts after the surgical treatment in the hospital and continues at home. Health professionals usually focus on the physical aspects and forget to maintain a holistic view, considering the spiritual needs of the patients, a crucial aspect in a disease of this significance. Some patients feel that health staff show little interest in their religiosity, barely exploring whether they need spiritual help.

Regarding the second theme (Table 4), on the psychosocial care provided by health professionals, two subthemes were identified: the role of each profession (six codes and 18 subcodes) and the reality of their day-to-day work (five codes and 21 subcodes). This theme contains the largest number of codes and subcodes.

Concerning the role of each profession, according to the opinion of nurses and physicians, the assessment of psychosocial needs in these patients can be performed by any professional and the alarm should be raised by anyone who detects unmet psychosocial needs, although nurses are the best suited professionals to identify PNs.

“Nursing. I think the nurse is the one who spends the most time with the patient and we are the ones who see those needs.” (Oncology nurse, hospital).

However, nurses themselves were aware of the importance of conducting a good nursing assessment that addresses this psychosocial sphere.

“Well, the social part isn't covered for me, no, it's not covered, neither during the admission nor outside, and… there are people or patients who are sad or angry, but you don't want to dig too much into why. So, if you can, it should be mandatory for me to address that. That area should be covered, just as their pain is covered. Because sometimes the pain can be because you don't manage it well, because of nerves or because of sadness, or even prolonging the stay because they are alone, because they are afraid. So, for me it's important, and many stays are lengthened because of social and psychological issues.” (Surgical nurse, hospital).

The role of physicians is primarily to refer patients with PNs to the psychologist and/or social worker. In addition, the characteristics of each professional were significant, depending on their sensitivity, previous experience, or empathy. In addition, it is necessary to have multidisciplinary teams, where the role of the psychologist/psycho-oncologist is key as the professional who can provide the main emotional support, followed by the social worker, although many professionals are unaware of their field of action.

Regarding the day-to-day reality, not enough importance is given to the psychosocial sphere during health care, although some professionals did believe that it is being adequately assessed (especially in primary care). The approach to the psychosocial sphere could be summarized as “No questions asked, no solution provided” or “It is only addressed once the more clinical part has been resolved.”

“What is life-threatening for this gentleman, the fact that he's sad or that he has colon cancer? Well, the cancer. You leave the other things aside, the sadness or how he copes with the tumor or his particular disease because first we're going to solve this and then we'll see.” (Surgeon, hospital).

In terms of the emotional needs of these patients, some professionals acknowledged that patients refuse psychiatric support because of the stigma toward mental health or the perception that they can cope on their own. However, the emotional sphere is generally neglected, and emotional needs are detected late.

During hospitalization, PNs or psychosocial risk is detected progressively, after several days of hospital stay and especially when hospital discharge is approaching. The professionals at the hospital stated that they are focused on physical needs and during the first years after diagnosis, the patient with cancer requires hospital care (i.e., surgery, treatments such as chemotherapy and radiotherapy, and checkups). Nonetheless, many professionals agreed that primary care would be the ideal setting for detecting and dealing with PNs, since they are more familiar with the patients' environment and family and social support network (neighbors, people in the community who can help them, volunteers, etc.). It was also noted that the approach to these PNs occurs in a social context where we live with our backs turned to the disease and death, where we should be more aware of the needs of patients and their social situation, as this often conditions their coping and even their treatment.

Concerning the third theme on the difficulties in dealing with PNs (Table 5), aspects related to the institution or the staff were highlighted. Three subthemes were included, with 10 codes and 6 subcodes. The main difficulties were the lack of training (conceived as voluntary and not favored during working hours) and lack of time. The lack of continuity between hospital and primary care or the scarce relevance of these issues in the clinical history was also noted. The professionals also identified problems of communication and interference with the family, as well as the risk of compassion fatigue, recognizing the fear of becoming too involved and the difficulty of providing emotional support.

“Not having a prescription to say: take this for two minutes, take it like this and come back tomorrow. To accompany, to be with the pain, with the doubt, and well, sometimes just to listen, other times you have to listen and… offer a solution.” (Physician, primary care).

The available resources related to psychosocial care were grouped into three subthemes and 11 codes (Table 5): information on resources (the patient is not informed about resources because the professionals themselves are unaware of them), the family as a psychosocial resource (the existing family support determines whether the person needs extra support or not), and the role of associations, which are providing psychological help and resources to cope with the major life change in cancer patients.

### 3.3. Distinctive Features of the Perception of Psychosocial Needs of Patients in Nursing and Medicine

The need to provide emotional support for cancer patients in nursing was one of the major aspects discussed. Thus, patients need to be able to talk and be listened to in order to face uncertainty and to confront death. The need for care was only mentioned by nurses, highlighting the importance of empathy on behalf of nurses and their need for support in certain situations (patients with ostomies). The physicians affirmed that not everyone needs psychological help, although patients find it difficult to accept the diagnosis.

Regarding the role of each profession, nurses reaffirmed that they perceived that they were the appropriate professional figure to detect PNs, especially during admission, whereas the physicians' role focused on therapeutic needs (surgical intervention, medical treatment, etc.), explicitly acknowledging their difficulty in addressing emotional needs.

In their day-to-day work, the nurses mostly felt that not enough importance is given to the psychosocial sphere, and therefore, unmet PNs are not attended to, especially at the emotional level, which coincides with the physicians' perception that they do not usually assess the patient's emotional state.

In relation to the difficulties in dealing with PNs, the nurses stated that they lacked the tools to avoid professional burnout, expressing the fear of becoming too involved with patients. They also acknowledged the difficulty of providing emotional support and the lack of professional sensitivity to the psychosocial sphere. Some physicians felt unable to address the patient's psychological experience, recognizing that they lack training going back to the time that they were residents, and acknowledging that they have an excessive workload. Physicians perceived that it would be easier to address this psychosocial sphere in primary care. They were aware of the importance of the psychosocial context and family support because these patients face a long process. Both professionals agreed on the need for training, especially during the workday.

In terms of resources, among the nurses, it was more frequent to refer the patient to social work for information on resources since they themselves were unaware of them. They also affirmed that associations are a support resource for dealing with the radical life change faced by patients, although for many nurses, it is an unknown resource, which they seldom recommend. Some physicians agreed that they could play a more proactive role in offering resources and barely mentioned the role of associations. Nurses acknowledged, to a greater extent, the difficult role of the family when one member has cancer, its value as an essential resource for patients, and the importance of considering a patient/family tandem.

### 3.4. Distinctive Features of the Perception of Psychosocial Needs of Patients in Hospital vs. Primary Care

The professionals referred t-hat at the hospital, many patients perceive at the onset that they do not need psychological support. Also, there is a sense that not all patients will need it. They are aware that the needs of these patients go beyond the physical sphere; however, according to Western medicine, most of the focus is placed on the physical sphere. Numerous referrals are made from the hospital to other services (especially from medicine to psychology and nursing to social work), and they demand that psychologists/psycho-oncologists should be a part of multidisciplinary teams. Hospital professionals perceived more professional burnout, more fear of compassion fatigue and lack of tools to face this situation. They also recognized the lack of time and excessive workload, which dehumanizes care, although they considered that a regular emotional assessment of patients is important as well as developing the skills to improve communication with patients and families. During hospital visits, professionals are better placed to assess the person's family support, whereas the socioeconomic situation of each family is more well known in primary care.

In contrast, in primary care, professionals emphasized the importance of the medical/nursing tandem, as well as the importance of assessing the circumstances in which patients live, who usually require more support than psychiatric treatment to detect vulnerable situations.

## 4. Discussion

Our results depict the professionals' perception regarding the PNs of patients with cancer and the role they can assume in responding to that aspect of care. These needs are mainly emotional, because of the impact of the diagnosis and the significance it may have on their future life. It is a tsunami at a personal and family level, where patients want to share their thoughts and emotions because they are afraid of losing their physical independence and are very concerned about uncertainty and death. They also have a need for information about the disease and about resources that they usually receive in associations or through social workers.

Nurses and physicians believe that each professional category has a different role, reflecting on the daily reality where various difficulties arise at the institutional level, together with a lack of time or training. Additionally, the information given to patients about available resources can be improved and the family represents an important resource.

The first category that emerged from the interviews with nurses and physicians was the needs of patients with cancer, who are undergoing a situation of great uncertainty in which they need psychosocial support, especially at the emotional level. According to other authors [2], emotional support is the main psychosocial need found in quantitative studies. Qualitative studies highlight the psychological and spiritual needs of patients related to feelings of fear, hopelessness, uncertainty about the future, sadness, anger, anxiety, frustration, and despair. The main support strategy of the professionals is active listening, empathy, and individual advice on how to cope with their situation. Many patients preferred this type of support to pharmacological treatment, which is what they usually receive [24].

Nurses are more likely to mention the need for patient support, whereas physicians feel that it may not be necessary in all cases [25]. Nurses perceive that patients want to share what they think and feel with them because they cannot always vent to their families and even if nurses or physicians are unable to solve their problems, they find it comforting to be listened to [2].

Professionals and patients coincided in their perception of cancer as a disease with limited chances of survival, which made them both feel vulnerable and in need of new knowledge and skills [14]. The professionals' own emotional vulnerability can make it difficult for them to cope with the requirements of a therapeutic relationship that lasts for years [25]. The importance of spiritual needs should also be recognized, a topic that is complex for nurses to address but critical for patients nearing the end of life. Again, active listening and normalizing conversations about their beliefs and respecting silences are successful communication strategies for providing spiritual care [26].

Concerning the psychosocial care provided by professionals, the respondents highlighted that the main role of nurses was the identification of PNs, whereas physicians oversaw the referral to other professionals, noting the advantage of working in multidisciplinary teams. In our study, physicians and nurses assumed the low importance given to the psychosocial sphere and when comparing their opinion, it was striking how nurses reaffirmed themselves as the main professional to detect PNs [9]. Other studies have also identified how nurses value the need to establish psychosocial care from the moment of diagnosis and accept the importance of their role yet frame it in the context of multidisciplinary teamwork [14]. The privileged role of nurses may be because good communication lays the foundations for a quality clinical relationship with patients and families [10]. In parallel, a systematic and comprehensive nursing assessment favors early detection and management of PNs [27]. It would be interesting to strive for a systematic detection of PNs with standardized and validated tools, such as the distress thermometer in cancer patients [28].

Regarding day-to-day work, in our study, the professionals stated that PNs are generally not assessed, especially during hospital admission where physical needs are prioritized. In particular, the physicians defended primary care as the most favorable environment to consider these needs and to gather information on the patient's environment, whereas primary care physicians and nurses emphasized teamwork between both professionals. The study by Easley et al. [13] found that both hospital and primary care physicians identified family physicians as the ideal professional to coordinate between different specialists, treat comorbidities, and inform and support patients and families. They pointed out the need to improve communication between levels and the training of family physicians. Other studies especially highlighted the role of the primary nurse in the care of patients who have survived cancer and benefit from a comprehensive long-term care model [15]. In general, the professionals appreciated having a figure to coordinate the care to whom both the patient and other care providers could turn to [25]. Also, having this professional as a reference throughout the process could favor the patient's confidence [29]. This longitudinal perspective is an important strength of public health systems with universal coverage and a network of multidisciplinary primary care teams such as that of Spain, in which the family doctor or nurse practitioner can be the professional of reference.

The third category was the difficulties in addressing psychosocial needs, emerging in relation to organization, lack of communication or continuity between levels and workload, or lack of time. In the hospital setting, workload and the lack of tools were of particular concern. In other studies, the lack of training in psychosocial care and communication skills, together with the high burden that prioritizes physical care over psychosocial care, are barriers to the hospital environment [9, 14]. In primary care, professionals identified certain difficulties such as low patient confidence, lack of time, and poor transmission of information from oncology to understand the patient's follow-up and play a more active role in their care [12]. However, the use of electronic medical records can be a strength to favor communication between levels of care and, therefore, their cooperation [29]. Workload increases due to the scarcity of resources (high patient/professional ratio) and also reduces the time available to provide quality care [29]. Thus, holistic care with the involvement of a multidisciplinary team with good communication between its members would favor the ability to address PNs [14].

While both physicians and nurses generally agreed on the difficulties, physicians placed more emphasis on the lack of training whereas nurses highlighted the fear of emotional exhaustion. In the hospital setting, there is more concern about compassion fatigue where involvement in psychosocial care can lead to emotional exhaustion [27]. Nonetheless, there are authors who emphasize the emotional implication [14] and teamwork [28] as a source of growth, professional satisfaction, and a protective factor against burnout. Additionally, in this environment, working in shifts worsens communication between professionals, and the high workload makes it difficult to deal with PNs because it requires time that may be unavailable.

The last category expressed by the professionals was information on available resources, with nurses relying especially on family and patient associations and physicians assuming that they should be more proactive in their advice on such resources. Regarding the setting, in the hospital, not all cases were considered to need a psychosocial assessment, with referral to a social worker or psychologist/psycho-oncologist when necessary, whereas in primary care, the professionals considered that they were capable of providing support without the need for referral. According to the literature consulted, the role of psycho-oncologists is highly appreciated by nurses and physicians; however, these professionals consider that few cases were referred to them, probably due to the stigma that patients feel toward psychological care and that referrals were made late [29]. Access can be complicated by problems such as waiting lists or noncoverage of this resource by the healthcare provider [25, 29]. Professionals value the family as a support resource from admission to care at discharge, appointments, and treatment plan and have an important role in patient groups and associations [29]. A systematic assessment of patients also helps to identify their family support network, and knowledge of their community assets helps to find resources to turn to for help.

From the point of view of nursing management, understanding the psychosocial needs of cancer patients places an additional demand on nursing staff. By acknowledging this, we underscore the importance of maintaining an appropriate patient-health professional ratio to ensure the delivery of comprehensive psychosocial care. Empowering nurses with the tools and resources to provide optimal psychosocial support can significantly impact the overall well-being of patients. There is evidence that assessing and addressing psychosocial needs in patients with cancer not only is effective but may also be cost saving, because the psychological problems oncological patients are associated with increased healthcare use, healthcare costs, and economic losses [30]. In addition to the cost effectiveness and cost-utility of psychosocial care, it can be hypothesized that the provision of psychosocial care can reduce both productivity losses and costs of providing informal care [31]. The provision of Psychiatric Mental Health Nurses consultation liaison services contributes to the detection and treatment of individuals with mental healthcare needs in nonmental healthcare settings as oncological services. Psychiatric advanced practice nurses are in the best position to identify the mental health needs of patients and enhance their health-related outcomes [32].

### 4.1. Limitations and Recommendations

This study has a number of limitations. Although we have had access to professionals (physicians and nurses) from various hospital and primary care services, it would be appropriate to incorporate other services (e.g., oncology day hospital or palliative care unit), considering the characteristics of the hospitals (hospitals >1000 beds vs. hospitals <200 beds) and private centers in order to have a more global view of the phenomenon. It would also be appropriate to continue this line of research by giving a voice to the patients themselves and their relatives in order to confirm our results. Another limitation is that only semistructured interviews were used for data collection. It would be advisable to complement this study by incorporating other qualitative methodologies such as participant observation or focus groups, and even to propose mixed-method studies with quantitative research.

## 5. Conclusions

According to the perspective of nurses and doctors, psychosocial needs are highly relevant in patients with cancer, as they often require emotional support to cope with a radical change in their lives. However, not enough professional importance is given, and most likely the emotional sphere is neglected because of the priority given to physical or therapeutic needs. The self-perceived role of nurses and physicians differs regarding PNs. Thus, nurses highlighted the need for these patients to receive emotional support and care whereas the role of physicians was more focused on referring these patients to other health professionals. The ideal network would be based on the primary care team as care coordinator and supported by multidisciplinary teams with specialized training, communication skills, time for comprehensive quality care, and information about resources. Through organizational measures and increased resources, the workload can be reduced to systematize the detection of PNs during the health professionals' daily work.

### 5.1. Implications for Nursing Management

Nurses claimed to be the ideal profession for the detection of psychosocial needs; however, it is necessary to improve nursing assessment and care plans and to give more importance to these needs in patients with cancer. Nursing management positions should be more sensitive to the time and training needs of nurses that can have a major impact on providing better care for people with cancer.

## Figures and Tables

**Table 1:** Semistructured interview guide.

The needs of patients with cancer	(1) In your experience with people with cancer, what kind of needs do you think they have?
	(2) When caring for cancer patients, what importance is given to psychosocial needs? How do you think this area is currently being assessed? What difficulties do you perceive in making this assessment?
	(3) Which professionals would be responsible for evaluating these psychosocial needs, at what level of care should this assessment be carried out (referring to specialized hospital care/primary care), have you made referrals to a psychologist/psychiatrist and/or social worker?
	(4) What training have you received on these topics?

Proposals for improving psychosocial care	(5) What is your opinion of the information given to these patients about resources or support services (psychology, social work)?
	(6) How do you think the detection of psychosocial needs in people with cancer can be improved?

**Table 2:** Characteristics of participants.

Code	Sex	Age	Profession	Clinical setting	Professional experience (years)	Psychosocial training	Family or friends with cancer
E1	Man	52	Registered nurse	Hospital (surgery)	32	No	Yes
E2	Woman	34	Registered nurse	Hospital (surgery)	12	No	Yes
E3	Woman	45	Registered nurse	Hospital (surgery)	20	No	Yes
E4	Woman	40	Registered nurse	Hospital (oncology)	17	Yes	No
E5	Woman	38	Registered nurse	Hospital (oncology)	14	No	Yes
E6	Woman	41	Registered nurse	Hospital (oncology)	20	No	No
E7	Man	44	Registered nurse	Hospital (oncology)	16	No	Yes
E8	Woman	60	Registered nurse	Hospital (oncology. Advance care planning nurse)	40	Yes	Yes
E9	Woman	60	Registered nurse	Primary care	43	No	Yes
E10	Woman	60	Registered nurse	Primary care	35	Yes	Yes
E11	Man	28	Registered nurse	Primary care	5	No	Yes
E12	Man	57	Registered nurse	Primary care	33	No	No
E13	Man	59	Registered nurse	Primary care	34	No	No
E14	Woman	54	Registered nurse	Primary care (palliative care)	30	Yes	No
E15	Woman	51	Physician	Hospital (surgery)	26	Yes	Yes
E16	Man	59	Physician	Hospital (surgery)	35	No	Yes
E17	Woman	29	Physician	Hospital (surgery resident)	5	No	No
E18	Woman	28	Physician	Hospital (otorhinolaryngology)	6	No	Yes
E19	Man	26	Physician	Hospital (otorhinolaryngology resident)	2	No	Yes
E20	Man	61	Physician	Hospital (oncology)	30	No	Yes
E21	Man	43	Physician	Hospital (oncology)	18	No	No
E22	Man	39	Physician	Hospital (oncology)	11	No	Yes
E23	Woman	60	Physician	Primary care	30	No	Yes
E24	Woman	60	Physician	Primary care	37	No	No
E25	Man	64	Physician	Primary care	41	No	No
E26	Man	59	Physician	Primary care (palliative care)	27	Yes	No

**Table 3:** The needs of patients with cancer from the professionals' perspective.

Themes	Subthemes	Codes
The needs of patients with cancer	The need for psychological support	They need emotional support
		They need emotional support throughout the process
		Initial perception of not needing psychological support
		They have significant affective needs
	Physical needs	Loss of functionality
		General (pain) and specific physical needs (depending on the type of tumor)
		Needs that transcend the physical sphere
		The psychosocial sphere is more of a priority than the physical sphere in people with cancer
	Need for information	They need information
		Only receiving the information they demand
		Improving information in the final stages of life
		There are still patients who are unaware of the diagnosis
	Speaking, sharing, and verbalizing fears	Facing death
		Overcoming the social stigma attached to cancer
		They need to talk, to be listened to
		We avoid talking about feelings
	Cancer as a tsunami	Coping with uncertainty
		It is hard to accept reality
		It is hard to come to terms with the diagnosis
		Each person experiences it in their own way
	The need for care	Information on organizational aspects
		Confusion due to erroneous information
		Respect for religious or spiritual needs
		Assimilating changes (ostomies)

**Table 4:** Psychosocial care provided by health professionals.

Themes	Subthemes	Codes	Subcodes
Psychosocial care provided by health professionals	The role of each profession	The assessment of social needs can be carried out by any professional and the alarm can be raised by anyone who detects it	
		Registered nurse	The most suited health professional to identify psychosocial needs
			We are not addressing the psychosocial sphere well within nursing assessment care plans
			The importance of a good nursing assessment
			They may refer patients to the social work unit
		Physician	Primarily refers patients with psychosocial needs to other healthcare providers
			They have difficulty addressing emotional needs
			They are focused on therapeutic needs
			The physician and registered nurse deliver patient care in tandem
			They would need to have a prior comprehensive assessment
		Influence of the characteristics of each professional	Psychosocial care depends on the sensitivity or previous experience of the health professional
			Psychosocial care is a personal decision of each professional
			Psychosocial care depends on the empathy of the professional
		The need for multidisciplinary teams	
		Expectations on psychology and social work	The importance of having psychologists/psycho-oncologists in the team
			The psychologist is the most appropriate professional to provide emotional support for patients
			The psychiatrist is not identified as a psychosocial support figure
			The social worker as a psychosocial support agent
			The main role of the social worker is to obtain resources
			Lack of knowledge of the role of social work
	Day-to-day reality	Addressing the psychosocial sphere during health care	It is not given enough importance among health professionals
			It is being adequately assessed
			It should explore the individual circumstances of each patient
			These are “nonmedical” support actions
			No questions asked, no solution provided
			It is addressed once the more clinical part has been resolved
		How are patients' emotional needs addressed?	Some patients refuse psychiatric support
			The emotional sphere is neglected
			We detect emotional needs too late
			Some patients avoid talking about emotions in consultation
			They often need more support than psychiatric treatment
		During the hospital stay	We cover the physical needs
			The patient with cancer is primarily cared for at hospital
			Social risk is detected throughout the hospital stay
			We detect social problems at discharge
		Primary care approach	It is the ideal setting for detecting and addressing psychosocial needs
			They have a better understanding of the patient's environment and support network
			They are there for and accompany the patient
		Health care for cancer patients in Spanish society	As a society, we live with our backs turned to illness and death
			The healthcare model should take patients' needs more into account
			The social situation conditions coping and treatment

**Table 5:** The challenges and resources for the psychosocial approach in patients with cancer.

Themes	Subthemes	Codes	Subcodes
Difficulties in addressing psychosocial needs	Institutional/staffing aspects	Lack of training	Psychosocial training on a voluntary basis
			Lack of training during specialized training
			Training during working hours is not favored
		Lack of time	We do not have enough time
			Assessing psychosocial aspects requires time for the patient to open up/be sincere
			Dehumanized care
		Lack of continuity of hospital/primary care	
		Minimal relevance in the electronical health record	
		Lack of professional sensitivity	
		Excessive workload	
	Communication problems	We should communicate better	
		Interferences with the family	
	Risk of compassion fatigue	Fear of getting too involved with the patient	
		It is difficult to provide emotional support	

The resources available for psychosocial care	Information on resources	The patient is not informed about resources	
		The professionals themselves are unaware of the resources	
		Some patients are aware of the resources and others are not	
	The family as a psychosocial resource	Consider the family's financial situation	
		Having family support (or not having it) determines whether or not you need extra support	
		The need to consider the patient/family tandem	
		The difficult role of the family	
		It is the patient's main emotional pillar	
	The role of associations	They receive psychological/other help from the associations	
		They provide resources to cope with the radical change of life	
		Associations as a resource are not proposed to patients	

## Data Availability

The data that support the findings of this study are available from the corresponding author upon reasonable request.
